# ABERRANT FUNCTIONAL CONNECTIVITY UNDERPINNING CHANGES IN LIFE-SPACE MOBILITY IN OLDER ADULTS

**DOI:** 10.1093/geroni/igz038.1763

**Published:** 2019-11-08

**Authors:** Chun Liang Hsu, John R Best, Rachel A Crockett, Patrick Chan, Teresa Liu-Ambrose

**Affiliations:** University of British Columbia, Vancouver, British Columbia, Canada

## Abstract

Subtle, but observable, changes in mobility often exist among older adults. Life-space mobility defines the frequency and extent of movements in the environment, and lower life-space mobility is associated with adverse health outcomes and mild cognitive impairment (MCI). Currently, the underlying mechanism of this association is not well understood. The aim of this study was to examine the functional neural correlates of reduced life-space mobility over 12 months. Thirty-five older adults over the age of 65 years with MCI were recruited and enrolled into this 12-month prospective study. Resting-state functional magnetic resonance imaging was completed at study baseline. Clinical assessment of anthropometric, behavioural measurements, and life-space mobility was conducted at study baseline and at the 12-month period. Over the 12-month study, the 35 participants demonstrated a significant reduction in LSA scores (paired sample t-test mean change=-6.53, p=0.01); greater baseline connectivity between the default mode network and the sensorimotor network was significantly associated with lower life-space mobility (R2=0.44, p=0.04). These findings suggest reduced life-space mobility in older adults may be partially due to altered inter-network connectivity in the brain such that normal neuro-cognitive motor behaviours is disrupted. Therefore, the maintenance of functional architecture of the brain may be essential in preserving the extent and frequency of older individuals’ movement in their environment.

